# A Comprehensive Evaluation of Potential Lung Function Associated Genes in the SpiroMeta General Population Sample

**DOI:** 10.1371/journal.pone.0019382

**Published:** 2011-05-20

**Authors:** Ma'en Obeidat, Louise V. Wain, Nick Shrine, Noor Kalsheker, Maria Soler Artigas, Emmanouela Repapi, Paul R. Burton, Toby Johnson, Adaikalavan Ramasamy, Jing Hua Zhao, Guangju Zhai, Jennifer E. Huffman, Veronique Vitart, Eva Albrecht, Wilmar Igl, Anna-Liisa Hartikainen, Anneli Pouta, Gemma Cadby, Jennie Hui, Lyle J. Palmer, David Hadley, Wendy L. McArdle, Alicja R. Rudnicka, Inês Barroso, Ruth J. F. Loos, Nicholas J. Wareham, Massimo Mangino, Nicole Soranzo, Tim D. Spector, Sven Gläser, Georg Homuth, Henry Völzke, Panos Deloukas, Raquel Granell, John Henderson, Ivica Grkovic, Stipan Jankovic, Lina Zgaga, Ozren Polašek, Igor Rudan, Alan F. Wright, Harry Campbell, Sarah H. Wild, James F. Wilson, Joachim Heinrich, Medea Imboden, Nicole M. Probst-Hensch, Ulf Gyllensten, Åsa Johansson, Ghazal Zaboli, Linda Mustelin, Taina Rantanen, Ida Surakka, Jaakko Kaprio, Marjo-Riitta Jarvelin, Caroline Hayward, David M. Evans, Beate Koch, Arthur William Musk, Paul Elliott, David P. Strachan, Martin D. Tobin, Ian Sayers, Ian P. Hall, SpiroMeta Consortium

**Affiliations:** 1 Nottingham Respiratory Biomedical Research Unit, Division of Therapeutics and Molecular Medicine, University Hospital of Nottingham, Nottingham, United Kingdom; 2 Departments of Health Sciences and Genetics, University of Leicester, Leicester, United Kingdom; 3 School of Molecular Medical Sciences and Centre for Genetics and Genomics, Queen's Medical Centre, University of Nottingham, Nottingham, United Kingdom; 4 Ludwig Institute for Cancer Research, University of Oxford, Oxford, United Kingdom; 5 Clinical Pharmacology, William Harvey Research Institute, Barts and The London School of Medicine and Dentistry, Queen Mary, University of London, London, United Kingdom; 6 Respiratory Epidemiology and Public Health Group, National Heart and Lung Institute, Imperial College London, London, United Kingdom; 7 Department of Epidemiology and Biostatistics, Imperial College London, London, United Kingdom; 8 MRC Epidemiology Unit, Institute of Metabolic Science, Cambridge, United Kingdom; 9 Department of Twin Research and Genetic Epidemiology, King's College London, London, United Kingdom; 10 MRC Human Genetics Unit, Institute of Genetics and Molecular Medicine, Western General Hospital, Edinburgh, Scotland, United Kingdom; 11 Institute of Genetic Epidemiology, Helmholtz Zentrum München, German Research Center for Environmental Health, Neuherberg, Germany; 12 Rudbeck Laboratory, Department of Genetics and Pathology, Uppsala University, Uppsala, Sweden; 13 Department of Clinical Sciences, Obstetrics and Gynecology, Institute of Clinical Medicine, University of Oulu, Oulu, Finland; 14 Department of Life Course and Services, National Institute for Health and Welfare, Oulu, Finland; 15 Ontario Institute for Cancer Research, Toronto, Canada; 16 Samuel Lunenfeld Research Institute, Toronto, Canada; 17 Molecular Genetics, PathWest Laboratory Medicine WA, Nedlands, Western Australia, Australia; 18 Busselton Population Medical Research Foundation, Sir Charles Gairdner Hospital, Nedlands, Western Australia, Australia; 19 Schools of Population Health and Pathology and Laboratory Medicine, University of Western Australia, Crawley, Australia; 20 Division of Community Health Sciences, St George's University of London, London, United Kingdom; 21 Pediatric Epidemiology Center, University of South Florida, Tampa, Florida, United States of America; 22 ALSPAC Laboratory, School of Social and Community Medicine, University of Bristol, Bristol, United Kingdom; 23 Wellcome Trust Sanger Institute, Cambridge, United Kingdom; 24 University of Cambridge Metabolic Research Labs, Institute of Metabolic Science Addenbrooke's Hospital Cambridge, Cambridge, United Kingdom; 25 Department of Internal Medicine B - Cardiology, Intensive Care, Pulmonary Medicine and Infectious Diseases, University of Greifswald, Greifswald, Germany; 26 Interfaculty Institute for Genetics and Functional Genomics, University of Greifswald, Greifswald, Germany; 27 Institute for Community Medicine, SHIP/Clinical-Epidemiological Research, University of Greifswald, Greifswald, Germany; 28 School of Social and Community Medicine, University of Bristol, Bristol, United Kingdom; 29 Croatian Centre for Global Health, The University of Split Medical School, Split, Croatia; 30 Andrija Stampar School of Public Health, Faculty of Medicine, University of Zagreb, Zagreb, Croatia; 31 Department of Public Health, University of Split, Split, Croatia; 32 Centre for Population Health Sciences, University of Edinburgh, Edinburgh, Scotland, United Kingdom; 33 Institute of Epidemiology I, Helmholtz Zentrum München, German Research Center for Environmental Health, Neuherberg, Germany; 34 University of Basel, Basel, Switzerland; 35 Swiss Tropical and Public Health Institute, Basel, Switzerland; 36 Rudbeck Laboratory, Department of Immunology, Genetics and Pathology, Uppsala University, Uppsala, Sweden; 37 Department of Public Health, University of Helsinki, Helsinki, Finland; 38 Department of Health Sciences and Gerontology Research Centre, University of Jyväskylä, Jyväskylä, Finland; 39 Institute for Molecular Medicine Finland FIMM, University of Helsinki, Helsinki, Finland; 40 National Institute for Health and Welfare, Helsinki, Finland; 41 Institute of Health Sciences, University of Oulu, Oulu, Finland; 42 Biocenter Oulu, University of Oulu, Oulu, Finland; 43 MRC Centre for Causal Analyses in Translational Epidemiology, School of Social and Community Medicine, University of Bristol, Bristol, United Kingdom; 44 Department of Respiratory Medicine, Sir Charles Gairdner Hospital, Nedlands, Western Australia, Australia; 45 Schools of Population Health and Medicine and Pharmacology, University of Western Australia, Crawley, Australia; 46 MRC-HPA Centre for Environment and Health, Imperial College London, London, United Kingdom; 47 SpiroMeta Consortium, Nottingham, Leicester, United Kingdom; University of Liverpool, United Kingdom

## Abstract

**Rationale:**

Lung function measures are heritable traits that predict population morbidity and mortality and are essential for the diagnosis of chronic obstructive pulmonary disease (COPD). Variations in many genes have been reported to affect these traits, but attempts at replication have provided conflicting results. Recently, we undertook a meta-analysis of Genome Wide Association Study (GWAS) results for lung function measures in 20,288 individuals from the general population (the SpiroMeta consortium).

**Objectives:**

To comprehensively analyse previously reported genetic associations with lung function measures, and to investigate whether single nucleotide polymorphisms (SNPs) in these genomic regions are associated with lung function in a large population sample.

**Methods:**

We analysed association for SNPs tagging 130 genes and 48 intergenic regions (+/−10 kb), after conducting a systematic review of the literature in the PubMed database for genetic association studies reporting lung function associations.

**Results:**

The analysis included 16,936 genotyped and imputed SNPs. No loci showed overall significant association for FEV_1_ or FEV_1_/FVC traits using a carefully defined significance threshold of 1.3×10^−5^. The most significant loci associated with FEV_1_ include SNPs tagging *MACROD2* (*P* = 6.81×10^−5^), *CNTN5* (*P* = 4.37×10^−4^), and *TRPV4* (*P* = 1.58×10^−3^). Among ever-smokers, *SERPINA1* showed the most significant association with FEV_1_ (*P* = 8.41×10^−5^), followed by *PDE4D* (*P* = 1.22×10^−4^). The strongest association with FEV_1_/FVC ratio was observed with *ABCC1* (*P* = 4.38×10^−4^), and *ESR1* (*P* = 5.42×10^−4^) among ever-smokers.

**Conclusions:**

Polymorphisms spanning previously associated lung function genes did not show strong evidence for association with lung function measures in the SpiroMeta consortium population. Common *SERPINA1* polymorphisms may affect FEV_1_ among smokers in the general population.

## Introduction

Pulmonary function is usually assessed by measurement of forced expiratory volume in one second (FEV_1_), forced vital capacity (FVC), and the ratio of FEV_1_ to FVC. The measurements are integral to the diagnosis of chronic obstructive pulmonary disease (COPD), and also are important long term predictors of population morbidity and mortality [1]. Reduced FEV_1_/FVC defines airways obstruction; whereas reduced FEV_1_ grades the severity of obstruction [2].

Pulmonary function is determined by both environmental and genetic factors. Tobacco smoking is the major environmental risk factor for the development of COPD. A genetic contribution to pulmonary function is well established with heritability estimates reaching 77 percent for FEV_1_ [3]. Linkage analyses within families have previously identified multiple genomic regions associated with spirometry measures and respiratory diseases. In addition, candidate gene studies have identified more than 100 genes which have been suggested to contribute to variability in lung function. The majority have been studied because of their potential pathophysiological role in the development of COPD. Some genes have been examined for association with lung function measurements in individuals with other specific respiratory diseases (most commonly asthma), or to a lesser extent, in the general population. With the exception of *SERPINA1*, which is the best documented genetic risk factor to influence the development of COPD [4], these genes have not shown consistent associations across different studies [5], [6].

Recently, we established the SpiroMeta consortium and published a large collective meta-analysis of lung function genome-wide association studies (GWAS) in 20,288 individuals of European origin, with follow-up of top SNPs in a further 54,276 individuals [7], [8]. Our study confirmed the hedgehog interacting protein (*HHIP*) association previously published [9], [10] and identified five new loci associated with FEV_1_ or FEV_1_/FVC ratio including tensin 1 gene (*TNS1*), glutathione S-transferase, C-terminal domain containing (*GSTCD*), 5-hydroxytryptamine receptor 4 (*HTR4*), advanced glycosylation end product-specific receptor (*AGER*), and thrombospondin, type I, domain containing 4 (*THSD4*). A study with similar design by the CHARGE consortium also identified *HHIP*, *AGER*, *HTR4*, and *GSTCD*, and in addition suggested a potential role of five additional genes (G protein-coupled receptor 126 (*GPR126*), a disintegrin and metalloproteinase domain 19 (*ADAM19*), family with sequence similarity 13, member A (*FAM13A*), patched homolog 1 (*PTCH1*), and phosphotyrosine interaction domain containing (*PID1*) [8].

The identification of these genes offers potential insight into the pathophysiology of altered lung function. The SpiroMeta consortium provides a powerful resource in which to study genetic associations with lung function. We aimed to comprehensively evaluate whether genes studied in candidate gene or small genome-wide association studies, and reported to be associated with lung function or COPD in these studies, were associated with lung function measures in this large general population sample.

## Results

### Literature search

The literature search identified 1719 publications. Of these, 104 reported one or more genetic associations: these are listed in text S1 in the online supporting information. These publications varied according to their study designs and the populations studied. 47 papers reported association with COPD using case control or family based designs. The remaining literature reported association with lung function traits within populations with specific respiratory diseases (asthma (26) and COPD (17)), or in general population cohorts (14). Nine publications studied other populations which included patients with cystic fibrosis (2), *SERPINA1* deficiency (2), cotton and grain workers (2), lung cancer (1), fire fighters (1) and post myocardial infarction (MI) patients (1). Some papers reported more than one endpoint.

These 104 relevant publications identified 130 genes and 48 intergenic SNPs. We investigated association between FEV_1_ and FEV_1_/FVC and each of the 16,936 genotyped and imputed SNPs spanning these regions in the SpiroMeta dataset.

### Contribution of all tested genes to lung function measures in SpiroMeta

**Figure 1:**
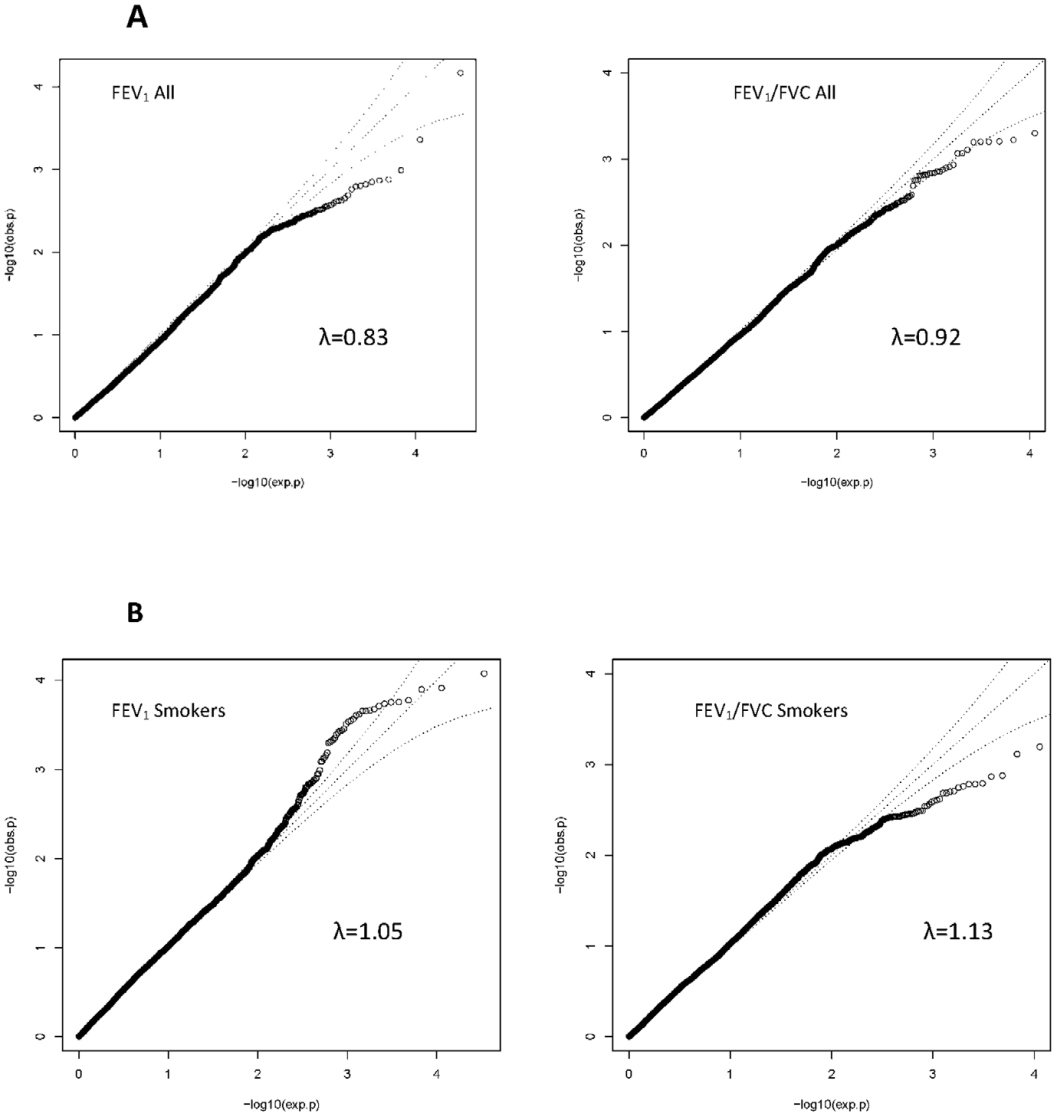
Quantile-quantile (Q-Q) plots of association results for FEV_1_ and FEV_1_/FVC. The Q-Q plot of association is shown for all individuals in Panel A, and separately for ever-smokers only in panel B. Q-Q plots compare the observed P values (obs.p) to expected P values (exp.p) on the logarithmic scale under the null hypothesis of no significant association. λ: Lambda.

Quantile-quantile (Q-Q) plots did not show large deviations between observed and expected P values for FEV_1_ and FEV_1_/FVC in all participants and for FEV_1_/FVC in ever-smokers (Figure 1). The plot of FEV_1_ in ever-smokers, however, shows slight deviations for high signal SNPs. The genomic inflation factor, λ for FEV_1_ is 0.83 in all individuals and 1.05 in smokers; λ for FEV_1_/FVC in all individuals is 0.92 and 1.13 in smokers.

Using the Bonferroni corrected P value threshold of 1.3×10^−5^, none of the tested SNPs demonstrated significant association with either FEV_1_ or FEV_1_/FVC.

### Association results in all individuals

In order to examine possible signals in greater detail, we also explored region plots for the top SNPs identified (SNPs with the lowest P values). The three top loci with the most significant P values for all regions tested in all individuals are presented in table 1.

**Table 1:** Association results for the three most significantly associated loci.

Gene	Locus	SNP	SNP function	coded allele	Coded allele frequency	N eff	Beta	Se	P
**FEV_1_ All individuals**									
***MACROD2***	20p12.1	rs204652	Intron	G	0.983	13551	−0.187	0.047	6.81×10^−5^
***CNTN5***	11q21-q22.2	rs17133553	Intron	T	0.966	13669	−0.100	0.029	4.37×10^−4^
***MTHFD1L***	6q25.1	rs803450	Intron	G	0.625	18497	0.036	0.011	1.03×10^−3^
**FEV_1_ Smokers**									
***SERPINA1***	14q32.13	rs3748312	Intron	T	0.167	9338	0.085	0.022	8.41×10^−5^
***PDE4D***	5q12	rs298028	Intron	T	0.283	10829	−0.069	0.018	1.22×10^−4^
***MACROD2***	20p12.1	rs204652	Intron	G	0.983	6872	−0.251	0.067	1.67×10^−4^
**FEV_1_/FVC All individuals**									
***ABCC1***	16p13.1	rs3887893	Intron	T	0.625	15509	−0.043	0.012	4.38×10^−4^
***ESR1***	6q25.1	rs11155818	Intron	G	0.992	12571	0.185	0.053	5.02×10^−4^
***CNTN5***	11q21-q22.2	rs1216170	Intron	C	0.284	18863	0.039	0.011	6.02×10^−4^
**FEV_1_/FVC Smokers**									
***ESR1***	6q25.1	rs9322335	Intron	T	0.217	9495	−0.061	0.018	5.42×10^−4^
***RHBDD1***	2q36.3	rs1864271	Intron	G	0.708	7385	0.066	0.019	6.40×10^−4^
***MTHFD1L***	6q25.1	rs1738567	Intron	C	0.367	10668	0.046	0.014	1.33×10^−3^

The table shows the three most significant loci associated with FEV_1_ and FEV_1_/FVC ratio in all individuals and ever-Smokers. N eff: the effective sample size. Beta: regression coefficient on a transformed scale. Se: standard error. P: *P* value. Coded allele frequency based on HapMap Release 24. *MACROD2:* MACRO domain containing 2. *CNTN5:* Contactin 5. *MTHFD1L* Methylenetetrahydrofolate dehydrogenase (NADP+ dependent) 1-like. *SERPINA1:* serpin peptidase inhibitor, clade A, member 1 (alpha-1 antitrypsin *AAT*). *PDE4D*: Phosphodiesterase 4D, cAMP-specific (phosphodiesterase E3 dunce homolog, Drosophila). *ABCC1*: ATP-binding cassette, sub-family C, member 1. *ESR1*: estrogen receptor 1. *RHBDD1*: rhomboid domain containing 1.

Among all individuals, the strongest association with FEV_1_ was with rs204652 in MACRO domain containing 2 (*MACROD2*) on chromosome 20. SNP rs17133553 in Contactin 5 (*CNTN5*) on chromosome 11 was the second top locus for association with FEV_1_ and third for FEV_1_/FVC ratio. SNP rs803450 in Methylenetetrahydrofolate dehydrogenase (NADP+ dependent) 1-like (*MTHFD1L*) on chromosome 6 showed association with FEV_1_ in all individuals. For FEV_1_/FVC ratio in all individuals the strongest association was with rs3887893 in ATP-binding cassette, sub-family C, member 1 (*ABCC1*) on chromosome 16, the second strongest signal was for rs11155818 in estrogen receptor 1(*ESR1*) on chromosome 6.

The region association plots around the most significant SNPs associated with FEV_1_ and FEV_1_/FVC in all individuals provide little evidence from supporting SNPs to suggest strong regions of association in *MACROD2*, *CNTN5*, *MTHFD1L*, and *ESR1*, and *ABCC1* in these data (See figure S1 in the online supporting information).

### Association results in ever-smokers

To study the impact of smoking on potential genetic associations with lung function, we repeated the analysis restricted to individuals who had ever smoked (ever-smokers). The most significant loci identified are shown in table 1.

Among ever-smokers, rs3748312 in serpin peptidase inhibitor, clade A, member 1 (*SERPINA1*) on chromosome 14, also known as alpha-1 antitrypsin (*AAT*) showed the strongest association with FEV_1_. SNP rs298028 in Phosphodiesterase 4D, cAMP-specific (phosphodiesterase E3 dunce homolog, Drosophila) (*PDE4D*) on chromosome 5 showed the second strongest association with FEV1, followed by MACROD2.

**Figure 2:**
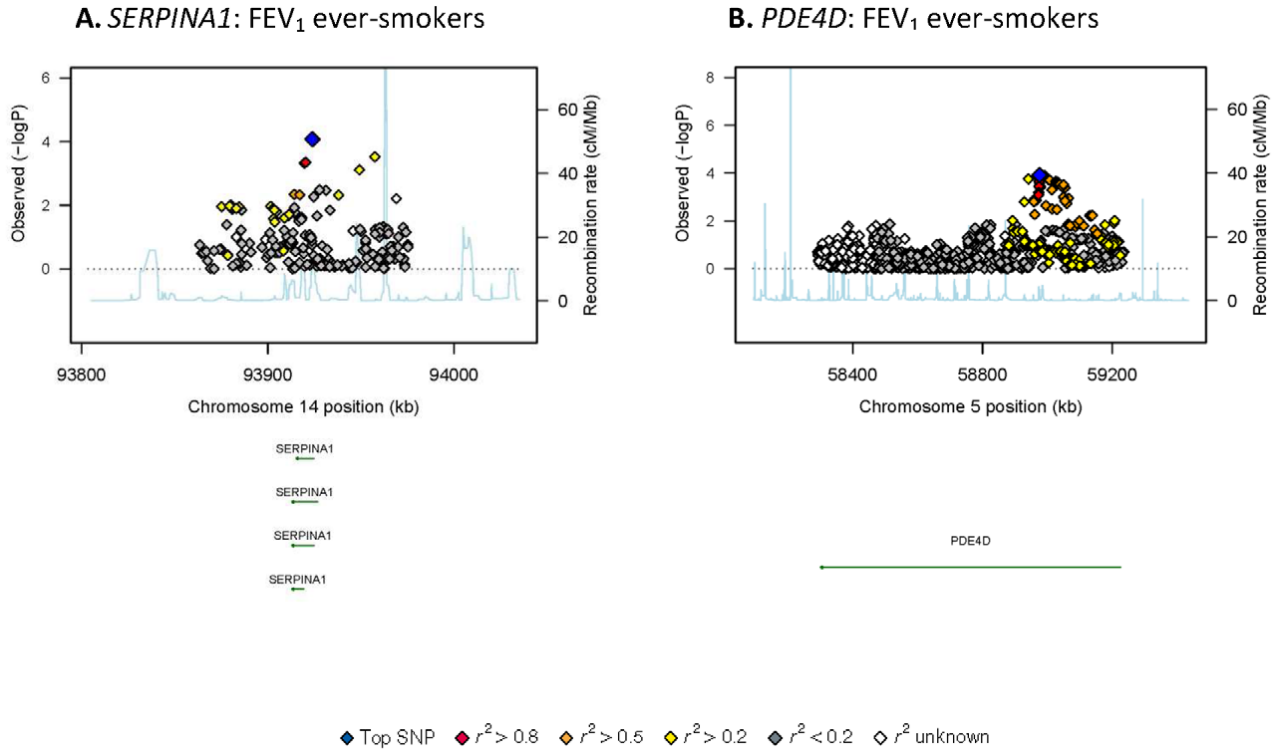
Regional association plots for *SERPINA1* (A) and *PDE4D* among ever-smokers (B) in SpiroMeta. Statistical significance of each SNP on the −log10 scale as a function of chromosome position (NCBI build 36). The sentinel SNP at each locus is shown in blue; the correlations (*r* ^2^) of each of the surrounding SNPs to the sentinel SNP are shown in the indicated colours. The relevant trait (FEV_1_ or FEV_1_/FVC ratio) is indicated for each plot. Recombination rate is shown in pale blue.

The strongest association with FEV1/FVC ratio among smokers was observed with rs9322335 in 1(*ESR1*) on chromosome 6. The second strongest association was rs1864271 in with rhomboid domain containing 1 (*RHBDD1*) on chromosome 2, followed by rs1738567 in (*MTHFD1L*) on chromosome 6. The region association plots for *SERPINA1* and *PDE4D* among ever-smokers (figure 2) show some supportive evidence for the association of these two loci. The region association plots for the additional loci among ever-smokers reported in table 1 are shown in figure S2 in the online supporting information.

### Association results excluding loci identified in previous GWAS

Because some of the regions identified were observed in the previously published small GWAS studies included in our literature search, we also present the top three genes for the relevant end points after excluding GWAS hits (Table 2).

**Table 2:** Association results for the most significant loci excluding genes identified in GWAS.

gene	locus	SNP	SNP function	coded allele	Coded allele Frequency	N eff	Beta	se	P
**FEV_1_ All**									
***PDE4D***	5q12	rs172362	Intron	C	0.133	18929	−0.048	0.015	1.36×10^−3^
***TRPV4***	12q24.1	rs3742030	Missense	G	0.992	17591	−0.110	0.035	1.58×10^−3^
***NAT2***	8p22	rs6988857	Intergenic	T	0.241	20080	0.035	0.011	2.37×10^−3^
**FEV_1_ Smokers**									
***SERPINA1***	14q32.13	rs3748312	Intron	T	0.167	9338	0.085	0.022	8.41×10^−5^
***PDE4D***	5q12	rs298028	Intron	T	0.283	20829	−0.069	0.018	1.22×10^−4^
***BCL2***	18q21.3	rs2850760	Intron	T	0.425	10888	0.050	0.014	4.66×10^−4^
**FEV_1_/FVC All**									
***ABCC1***	16p13.1	rs3887893	Intron	T	0.625	15509	−0.043	0.012	4.38×10^−4^
***ESR1***	6q25.1	rs11155818	Intron	G	0.992	12571	0.185	0.053	5.02×10^−4^
***AIF1***	6p21.3	rs3132451	5′near gene	G	0.883	19889	0.045	0.014	7.84×10^−4^
**FEV_1_/FVC Smokers**									
***ESR1***	6q25.1	rs9322335	Intron	T	0.217	9495	−0.061	0.018	5.42×10^−4^
***ABCC1***	16p13.1	rs3887893	Intron	T	0.625	8156	−0.054	0.017	1.36×10^−3^
***CD22***	19q13.1	rs7251526	Intron	T	0.246	8328	−0.054	0.018	2.00×10^−3^

The table shows association results for the three most significant loci associated with FEV_1_ and FEV_1_/FVC, in all individuals and in ever-smokers after excluding genes identified in GWAS. N eff: the effective sample size. Beta: regression coefficient on a transformed scale. Se: standard error. P: P value. Coded allele frequency based on HapMap Release 24. *PDE4D*: Phosphodiesterase 4D, cAMP-specific (phosphodiesterase E3 dunce homolog, Drosophila). *TRPV4:* transient receptor potential cation channel, subfamily V, member 4. *NAT2:* N-acetyltransferase 2. *SERPINA1:* serpin peptidase inhibitor, clade A, member 1 (alpha-1 antitrypsin *AAT*). *BCL2:* B-cell CLL/lymphoma 2. *ABCC1*: ATP-binding cassette, sub-family C, member 1. *AIF1:* allograft inflammatory factor 1. *ESR1*: estrogen receptor 1. *CD22:* cluster of differentiation; CD22 molecule.

The additional genes identified in this analysis for association with FEV_1_ among all individuals were the transient receptor potential cation channel, subfamily V, member 4 (*TRPV4*) on chromosome 12, and N-acetyltransferase 2 (*NAT2*) on chromosome 8. Among ever-smokers, association results for FEV_1_ identified B-cell CLL/lymphoma 2 (*BCL2*) on chromosome 18. Association results for FEV_1_/FVC ratio identified allograft inflammatory factor 1(*AIF1*) on chromosome 6 among all individuals, and cluster of differentiation; CD22 molecule (*CD22*) on chromosome 19 among ever-smokers. The region association plots for the most significant loci in table 2 and not presented earlier are shown in figure S3 in the online supporting information. The plots show some additional support for all presented loci except for *ABCC1* among ever-smokers.

## Discussion

In the SpiroMeta study, we generated a comprehensive dataset to analyse associations between genetic variants and lung function in the general population [7]. There have been many small previous studies, mostly of individual candidate genes examining association with lung function, which have produced conflicting results. Therefore, in this paper, we undertook a comprehensive literature review to identify relevant gene regions and analysed potential associations with FEV_1_ and FEV_1_/FVC ratio in all individuals within SpiroMeta. In addition, given the impact of smoking on lung function, we also analysed the associations separately in ever-smokers. There were no strong association signals in never-smokers group (data available on request).

The main conclusion from this study is that, within 178 previously reported regions, we found no SNP associations which exceeded the significance threshold (P<1.3×10^−5^) we employed after correction for multiple testing. Our results suggest these regions do not constitute major genetic determinants of lung function measures at the general population level. The lack of replication and sometimes contradicting results in previous studies may reflect the fact that many previously reported associations came from studies with small sample sizes, possibly leading to false positive results.

Despite the failure to identify any overall significant contribution of a single SNP from previously reported genes to lung function, there are some potentially interesting signals apparent from the region plots suggesting that there may be a small signal from variants in some of the genes of interest.

*SERPINA1* showed the strongest association with FEV_1_ among smokers (8.41×10^−5^). It encodes alpha-1 Antitrypsin protein (AAT), mainly produced in the liver and has the primary role of inhibiting neutrophil elastase in the lungs [11]. Protein variants of this gene have been classified based on their migration in an isoelectric pH gradient from A to Z. Among Caucasians, the M allele is the most common allele with six subtypes: M1–M6 with allele frequencies greater than 95 percent and associated with normal AAT levels. The common deficiency variants; S (frequency 0.02–0.03) and Z (frequency 0.01–0.03), are associated with mild and severe reductions in serum AAT levels, respectively [11], [12]. The *r* ^2^ between the Z allele rs28929474 and rs3748312 is 0.08 (based on 1000 Genomes Project pilot 1 data from 120 CEU individuals). Our top SNP, rs3748312, is in LD (*r* ^2^ = 0.603) with the M1 allele SNP rs6647, but is in very weak LD with M2 rs709932 (*r* ^2^ = 0.033) and M3 rs1303 (*r* ^2^ = 0.051). The S allele SNP rs45551939 (merged into rs17580) was not found in HapMap (version24). It is possible that the signal observed in our data is due to variants with effects on gene expression and/or protein levels, and this idea is supported by a previous study showing novel variants in *SERPINA1* to be associated with increased susceptibility to COPD independently of the Z allele [13]. The relatively strong signal observed in our study suggests a possible role for variants in *SERPINA1* in smokers at the general population level beyond that observed in carriers of known deficient alleles.

The *PDE4D* gene encodes the type 4D phosphodiesterase, which degrades cyclic adenosine monophosphate (cAMP), an important signal transduction molecule in all cell types. Polymorphisms within *PDE4D* have been associated with stroke [14], and bone mineral density [15]. *PDE4D* is the most dominant phosphodiesterase in the lungs and plays an important role in regulating airway smooth muscle contractility [16] demonstrated by *PDE4D* knockout mice lacking response to methacholine [17]. A study in a Japanese population reported association of one *PDE4D* SNP (rs829259) and a haplotype consisting of rs10075508 and one interleukin 13 (*IL13*) SNP with COPD [18]. SNP rs829259 was not associated with FEV_1_ in all individuals (P = 0.68) and in smokers (P = 0.21) in our study, and SNP rs10075508 was not genotyped or imputed in SpiroMeta. A recent GWAS has also identified *PDE4D* as an asthma susceptibility gene [19], however, none of the top 5 SNPs associated with asthma is present in our dataset, and the linkage disequilibrium (LD) with SNPs in SpiroMeta is low, so it is difficult to comment on their contribution to lung function measures in our study.

Our study has a number of strengths. First, we have power to detect associations of small magnitude, with data on 20,288 individuals from 14 European studies with more than 2.5 million genotyped and imputed SNPs. Second, we aimed to minimise Type 1 error whilst taking appropriate account of the correlation between neighbouring SNPs. Finally, the literature search was designed to be comprehensive to include all reported genetic variants with effect on lung function irrespective of disease status or ethnicity. To our knowledge, this is the first study to comprehensively evaluate the role of previously associated genes in a large genome-wide association study.

However, it is important to recognise the limitations of our study. We have tested for association in a general population sample; the magnitude of effect of these genetic variants may be greater in populations enriched with individuals with respiratory diseases such as asthma and COPD. Second, we have tested with cross sectional lung function measures. Some of the variants tested might affect longitudinal changes by accelerating or decelerating the decline in lung function, although this would still be expected to result in effects evident in cross sectional data. Third, the power of our study to detect associations of SNPs with modest effect sizes on lung function was limited given our relatively conservative approach to multiple testing, therefore we cannot rule out a real but modest effect of some of these loci on lung function and susceptibility to respiratory diseases in the general population. Alternative approaches could be to utilise a priori evidence about the reported direction of effect and a priori assumptions about the likely presence of multiple causal variants. Fourth, we tested for association with lung function measures among individuals of European ancestry, and the contribution of these variants to lung function in other populations may vary. Finally, the coverage of tested genetic regions varies depending on the genome-wide arrays used and imputation quality metrics.

In conclusion, we have shown that none of the SNPs tagging the genes previously reported to determine lung function were significantly associated with FEV_1_ or FEV_1_/FVC ratio in the SpiroMeta general population study. We found some evidence to suggest a possible contribution for the *SERPINA1* and *PDE4D* loci to lung function in smokers which warrant further study. As a resource to the scientific community we have provided the complete association results (Dataset S1) in the online supporting information.

## Methods

### Systematic Literature search

We conducted a literature search in PubMed in October 2009 for genetic association studies of lung function measurements and/or COPD. The search terms used were: “Lung function” OR “pulmonary function” OR “FEV_1_” OR “FEV_1_/FVC” OR “Forced Expiratory Volume” OR “Forced Vital Capacity” OR “ASTHMA” OR “COPD” OR “BRONCHIAL HYPERRESPONSIVENESS” OR “BHR” OR “obstructive lung disease” AND “SNP” OR “single nucleotide polymorphism” OR “polymorphism” OR “gene” OR “genetic” OR “genom*” OR “variation” AND “Linkage” OR “association”

From the search results, we included relevant papers reporting only positive association results. For the three GWAS papers identified, we took a more inclusive approach and included all loci presented in the publication body, and not just those meeting genome-wide significance. We excluded papers reporting associations with respiratory diseases (e.g. asthma) without association with lung function measurements.

### Statistical analysis

The genes and intergenic SNPs identified in the relevant literature were evaluated in the SpiroMeta dataset using an extended region of +/−10 kilobases (kb) from the gene coordinates downloaded from the UCSC genome browser (we used the SNP coordinate +/−10 kb for intergenic SNPs). Meta-analysis association results for SNPs in these (+/−10 kb extended) regions were extracted from the SpiroMeta dataset for both FEV_1_ and FEV_1_/FVC in all individuals and separately in ever–smokers. The complete cohort descriptions, study design and methods have been previously reported [7], but we provide here a brief summary. At study level, non-genotyped SNPs were imputed using standard approaches [18], [20] to facilitate meta-analysis of studies employing different genotyping platforms. Thus up to 2,705,257 SNPs were tested for association with FEV1 and FEV1/FVC using additive models and adjusting for age, sex, height and ancestry principal components. Then, the results were meta-analysed across studies using inverse variance weighting. Genomic control was applied at the study level and after the meta-analysis to correct for test inflation due to population stratification [21]. We excluded SNPs which were not well measured or imputed in the study (identifiable by an “effective sample size” of <50% of the total sample size) [7]. In all, we identified 16,936 genotyped and imputed SNPs in the gene and intergenic regions described above which met our inclusion criteria.

In order to correct for multiple testing of SNPs in linkage disequilibrium we used Li and Ji's [22] method for calculating the effective number of independent tests from pairwise SNP correlations. Pairwise SNP correlations were obtained from reference genotypes of 1468 subjects in the Busselton study [23]. We estimated that the association tests for the 16,936 highly correlated SNPs we selected in the regions of interest equated to 3,891 independent tests.

To maintain a Type 1 error rate of 5%, we adjusted the significance threshold using a Bonferroni correction (0.05/3891). Thus a threshold of 1.3×10^−5^ was used to determine statistical significance.

## Supporting Information

Figure S1 Regional association plots of the most significant lung function–associated loci among all individuals in SpiroMeta (A–F). Statistical significance of each SNP on the −log10 scale as a function of chromosome position (NCBI build 36). The sentinel SNP at each locus is shown in blue; the correlations (*r* ^2^) of each of the surrounding SNPs to the sentinel SNP are shown in the indicated colours. The relevant trait (FEV_1_ or FEV_1_/FVC ratio) is indicated for each plot. Recombination rate is shown in pale blue.
(TIFF)

Figure S2 Regional association plots of the most significant lung function–associated loci among ever-smokers in SpiroMeta (A–D). Statistical significance of each SNP on the −log10 scale as a function of chromosome position (NCBI build 36). The sentinel SNP at each locus is shown in blue; the correlations (*r* ^2^) of each of the surrounding SNPs to the sentinel SNP are shown in the indicated colours. The relevant trait (FEV_1_ or FEV_1_/FVC ratio) is indicated for each plot. Recombination rate is shown in pale blue.
(TIF)

Figure S3 Regional association plots of the most significant lung function–associated loci (A–G) after excluding genes identified in GWAS. Statistical significance of each SNP on the −log10 scale as a function of chromosome position (NCBI build 36). The sentinel SNP at each locus is shown in blue; the correlations (*r* ^2^) of each of the surrounding SNPs to the sentinel SNP are shown in the indicated colours. The relevant trait (FEV_1_ or FEV_1_/FVC ratio) and whether it is in all individuals or ever-smokers is indicated for each plot. Recombination rate is shown in pale blue.
(TIF)

Text S1 The 104 relevant publications identified in the literature search.
(DOC)

Dataset S1 Complete FEV_1_ and FEV_1_/FVC association results for all individuals and separately for ever-smokers.
(XLS)

## References

[1] Schunemann HJ, Dorn J, Grant BJ, Winkelstein W, Trevisan M (2000). Pulmonary function is a long-term predictor of mortality in the general population: 29-year follow-up of the Buffalo Health Study.. Chest.

[2] Rabe KF, Hurd S, Anzueto A, Barnes PJ, Buist SA (2007). Global strategy for the diagnosis, management, and prevention of chronic obstructive pulmonary disease: GOLD executive summary.. American Journal of Respiratory & Critical Care Medicine.

[3] Hubert HB, Fabsitz RR, Feinleib M, Gwinn C (1982). Genetic and environmental influences on pulmonary function in adult twins.. American Review of Respiratory Disease.

[4] Laurell C-B, Eriksson S (1963). The Electrophoretic alpha 1-Globulin Pattern of Serum in alpha 1-Antitrypsin Deficiency.. Scandinavian Journal of Clinical and Laboratory Investigation.

[5] Hersh CP, Demeo DL, Lange C, Litonjua AA, Reilly JJ (2005). Attempted replication of reported chronic obstructive pulmonary disease candidate gene associations.. American Journal of Respiratory Cell & Molecular Biology.

[6] Smolonska J, Wijmenga C, Postma DS, Boezen HM (2009). Meta-analyses on suspected chronic obstructive pulmonary disease genes: a summary of 20 years' research.. American Journal of Respiratory & Critical Care Medicine.

[7] Repapi E, Sayers I, Wain LV, Burton PR, Johnson T (2010). Genome-wide association study identifies five loci associated with lung function.. Nature Genetics.

[8] Hancock DB, Eijgelsheim M, Wilk JB, Gharib SA, Loehr LR (2010). Meta-analyses of genome-wide association studies identify multiple loci associated with pulmonary function.. Nature Genetics.

[9] Wilk JB, Chen T-H, Gottlieb DJ, Walter RE, Nagle MW (2009). A genome-wide association study of pulmonary function measures in the Framingham Heart Study.. PLoS Genetics.

[10] Pillai SG, Ge D, Zhu G, Kong X, Shianna KV (2009). A genome-wide association study in chronic obstructive pulmonary disease (COPD): identification of two major susceptibility loci.. PLoS Genetics.

[11] Kalsheker NA (2009). alpha1-Antitrypsin deficiency: best clinical practice.. Journal of Clinical Pathology.

[12] DeMeo DL, Silverman EK (2004). Alpha1-antitrypsin deficiency. 2: genetic aspects of alpha(1)-antitrypsin deficiency: phenotypes and genetic modifiers of emphysema risk.. Thorax.

[13] Chappell S, Daly L, Morgan K, Guetta Baranes T, Roca J (2006). Cryptic haplotypes of SERPINA1 confer susceptibility to chronic obstructive pulmonary disease.. Human Mutation.

[14] Gretarsdottir S, Thorleifsson G, Reynisdottir ST, Manolescu A, Jonsdottir S (2003). The gene encoding phosphodiesterase 4D confers risk of ischemic stroke.[Erratum appears in Nat Genet. 2005 May;37(5):555].. Nature Genetics.

[15] Reneland RH, Mah S, Kammerer S, Hoyal CR, Marnellos G (2005). Association between a variation in the phosphodiesterase 4D gene and bone mineral density.. BMC Medical Genetics.

[16] Mehats C, Jin SLC, Wahlstrom J, Law E, Umetsu DT (2003). PDE4D plays a critical role in the control of airway smooth muscle contraction.. FASEB Journal.

[17] Hansen G, Jin S, Umetsu DT, Conti M (2000). Absence of muscarinic cholinergic airway responses in mice deficient in the cyclic nucleotide phosphodiesterase PDE4D.. Proceedings of the National Academy of Sciences of the United States of America.

[18] Homma S, Sakamoto T, Hegab AE, Saitoh W, Nomura A (2006). Association of phosphodiesterase 4D gene polymorphisms with chronic obstructive pulmonary disease: relationship to interleukin 13 gene polymorphism.. International Journal of Molecular Medicine.

[19] Himes BE, Hunninghake GM, Baurley JW, Rafaels NM, Sleiman P (2009). Genome-wide association analysis identifies PDE4D as an asthma-susceptibility gene.. American Journal of Human Genetics.

[20] Marchini J, Howie B, Myers S, McVean G, Donnelly P (2007). A new multipoint method for genome-wide association studies by imputation of genotypes.. Nat Genet.

[21] Devlin B, Roeder K (1999). Genomic control for association studies.. Biometrics.

[22] Li J, Ji L (2005). Adjusting multiple testing in multilocus analyses using the eigenvalues of a correlation matrix.. Heredity.

[23] Palmer LJ, Knuiman MW, Divitini ML, Burton PR, James AL (2001). Familial aggregation and heritability of adult lung function: results from the Busselton Health Study.. European Respiratory Journal.

