# Supplementary figures and images for: A Comprehensive Evaluation of Potential Lung Function Associated Genes in the SpiroMeta General Population Sample
Source: PLoS One. 2011 May 20;6(5):e19382. doi: 10.1371/journal.pone.0019382 (PMC3098839; doi:10.1371/journal.pone.0019382)

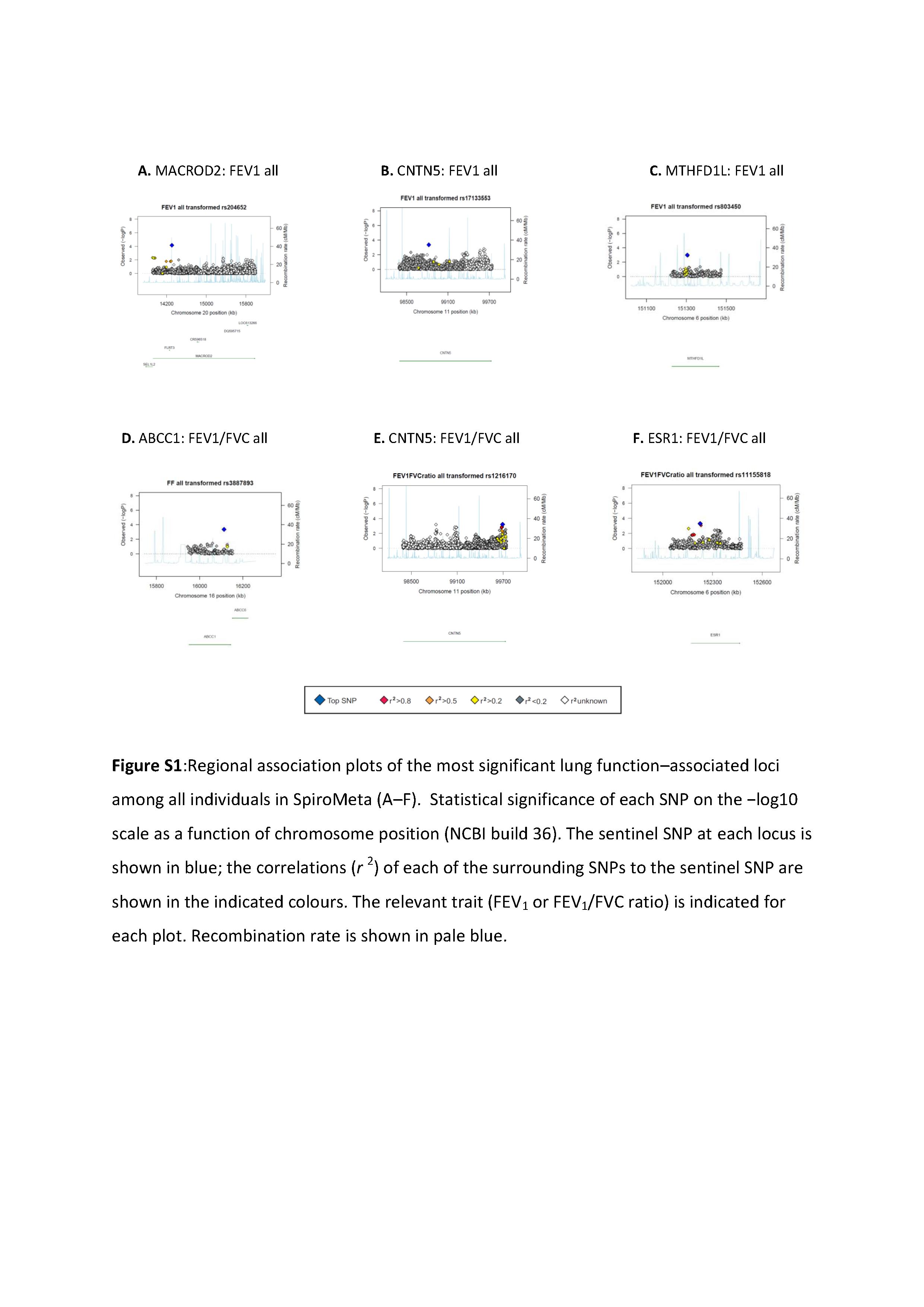

Supplement: Figure S1 — Regional association plots of the most significant lung function–associated loci among all individuals in SpiroMeta (A–F). Statistical significance of each SNP on the −log10 scale as a function of chromosome position (NCBI build 36). The sentinel SNP at each locus is shown in blue; the correlations (r 2) of each of the surrounding SNPs to the sentinel SNP are shown in the indicated colours. The relevant trait (FEV1 or FEV1/FVC ratio) is indicated for each plot. Recombination rate is shown in pale blue. (TIFF) [file pone.0019382.s001.tiff]

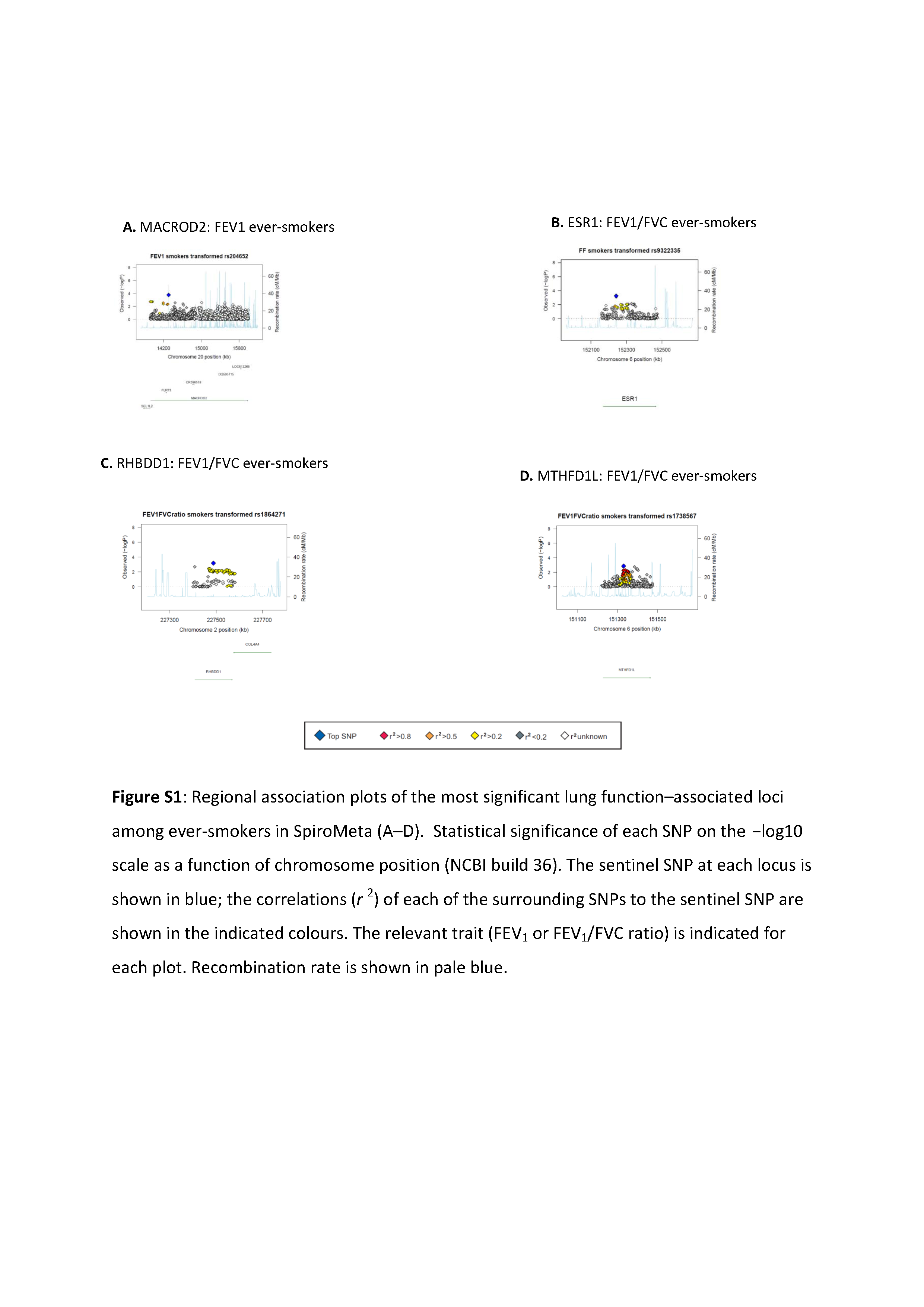

Supplement: Figure S2 — Regional association plots of the most significant lung function–associated loci among ever-smokers in SpiroMeta (A–D). Statistical significance of each SNP on the −log10 scale as a function of chromosome position (NCBI build 36). The sentinel SNP at each locus is shown in blue; the correlations (r 2) of each of the surrounding SNPs to the sentinel SNP are shown in the indicated colours. The relevant trait (FEV1 or FEV1/FVC ratio) is indicated for each plot. Recombination rate is shown in pale blue. (TIF) [file pone.0019382.s002.tif]

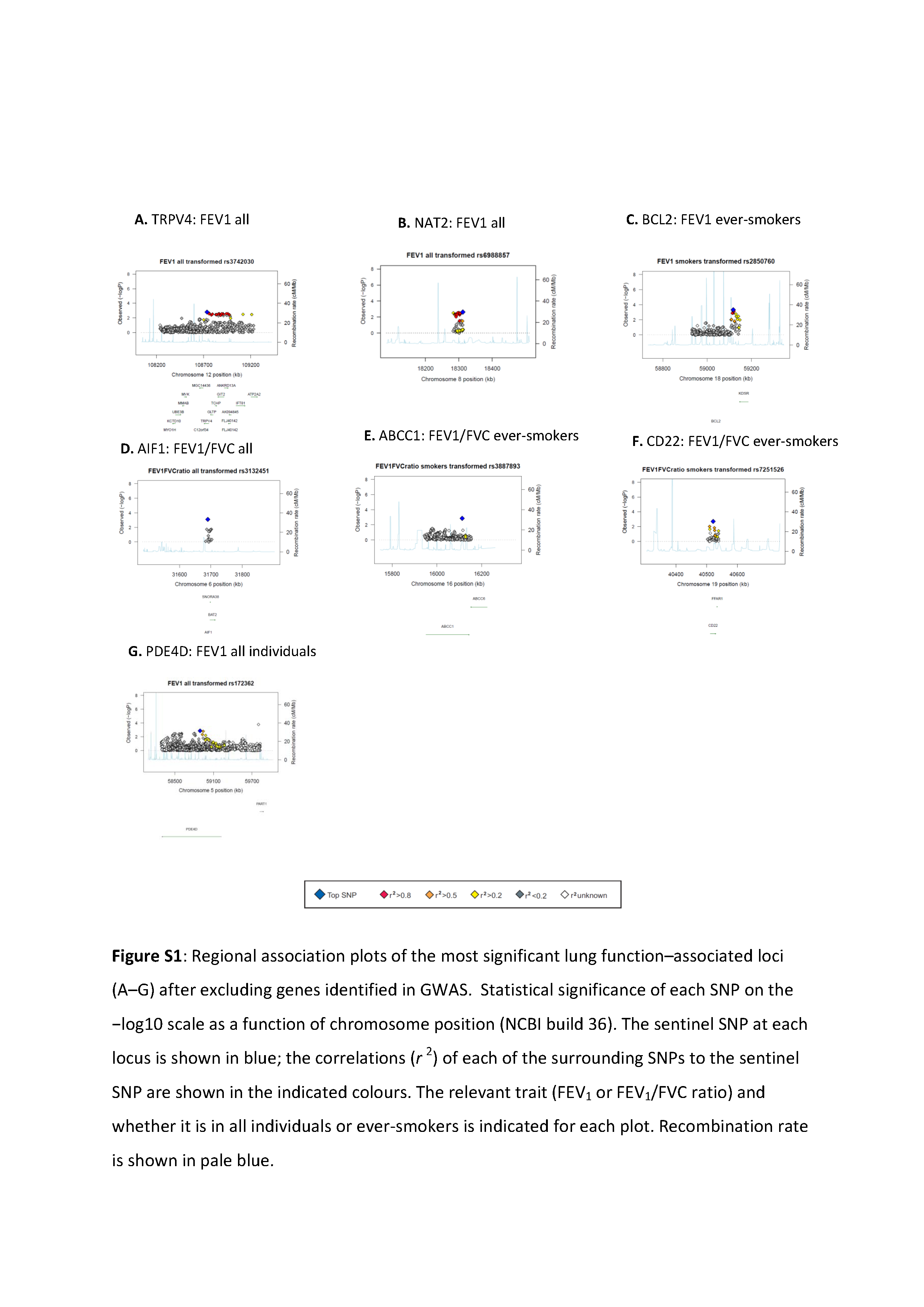

Supplement: Figure S3 — Regional association plots of the most significant lung function–associated loci (A–G) after excluding genes identified in GWAS. Statistical significance of each SNP on the −log10 scale as a function of chromosome position (NCBI build 36). The sentinel SNP at each locus is shown in blue; the correlations (r 2) of each of the surrounding SNPs to the sentinel SNP are shown in the indicated colours. The relevant trait (FEV1 or FEV1/FVC ratio) and whether it is in all individuals or ever-smokers is indicated for each plot. Recombination rate is shown in pale blue. (TIF) [file pone.0019382.s003.tif]
