# Supplementary material for: A Comprehensive Evaluation of Potential Lung Function Associated Genes in the SpiroMeta General Population Sample
Source: PLoS One. 2011 May 20;6(5):e19382. doi: 10.1371/journal.pone.0019382 (PMC3098839; doi:10.1371/journal.pone.0019382)
Supplement: Text S1 — The 104 relevant publications identified in the literature search. (DOC) [file pone.0019382.s004.doc]

**Comprehensive evaluation of lung function associated genes in the SpiroMeta general population study**

***Supporting information Text S1***

The 104 relevant publications identified in the literature search are presented below [1-104] :

1. Siedlinski, M., H. Boezen, J.M.A. Boer, H.A. Smit, and D.S. Postma, *ABCC1 polymorphisms contribute to level and decline of lung function in two population-based cohorts.* Pharmacogenet Genomics, 2009. **19**(9): p. 675-84.

2. Sadeghnejad, A., J.A. Ohar, S.L. Zheng, D.A. Sterling, G.A. Hawkins, D.A. Meyers, and E.R. Bleecker, *Adam33 polymorphisms are associated with COPD and lung function in long-term tobacco smokers.* Respir Res, 2009. **10**: p. 21.

3. Seibold, M.A., B. Wang, C. Eng, G. Kumar, K.B. Beckman, S. Sen, S. Choudhry, K. Meade, M. Lenoir, H.G. Watson, S. Thyne, L.K. Williams, R. Kumar, K.B. Weiss, L.C. Grammer, P.C. Avila, R.P. Schleimer, E.G. Burchard, and R. Brenner, *An african-specific functional polymorphism in KCNMB1 shows sex-specific association with asthma severity.* Hum Mol Genet, 2008. **17**(17): p. 2681-90.

4. Hui, J., L.J. Palmer, A.L. James, A.W. Musk, and J.P. Beilby, *AluyMICB dimorphism within the class I region of the major histocompatibility complex is associated with asthma and airflow obstruction in the Busselton population.* Clin Exp Allergy, 2006. **36**(6): p. 728-34.

5. He, J.-Q., J. Ruan, J.E. Connett, N.R. Anthonisen, P.D. Pare, and A.J. Sandford, *Antioxidant gene polymorphisms and susceptibility to a rapid decline in lung function in smokers.* Am J Respir Crit Care Med, 2002. **166**(3): p. 323-8.

6. Hegab, A.E., T. Sakamoto, Y. Uchida, A. Nomura, Y. Ishii, Y. Morishima, M. Mochizuki, T. Kimura, W. Saitoh, T. Kiwamoto, T. Iizuka, H.H. Massoud, H.M. Massoud, K.M. Hassanein, and K. Sekizawa, *Association analysis of tissue inhibitor of metalloproteinase2 gene polymorphisms with COPD in Egyptians.* Respir Med, 2005. **99**(1): p. 107-10.

7. Al-Abdulhadi, S.A. and M.W. Al-Rabia, *Association and preferential transmission of the CCR2V64I polymorphism with absence of asthma in high-risk families.* Saudi Med J, 2008. **29**(12): p. 1711-8.

8. Burchard, E.G., E.K. Silverman, L.J. Rosenwasser, L. Borish, C. Yandava, A. Pillari, S.T. Weiss, J. Hasday, C.M. Lilly, J.G. Ford, and J.M. Drazen, *Association between a sequence variant in the IL-4 gene promoter and FEV(1) in asthma.* Am J Respir Crit Care Med, 1999. **160**(3): p. 919-22.

9. Ishii, T., T. Matsuse, S. Teramoto, H. Matsui, T. Hosoi, Y. Fukuchi, and Y. Ouchi, *Association between alpha-1-antichymotrypsin polymorphism and susceptibility to chronic obstructive pulmonary disease.* Eur J Clin Invest, 2000. **30**(6): p. 543-8.

10. Leung, T.F., I.H.S. Chan, G.W.K. Wong, C.Y. Li, N.L.S. Tang, E. Yung, and C.W.K. Lam, *Association between candidate genes and lung function growth in Chinese asthmatic children.* Clin Exp Allergy, 2007. **37**(10): p. 1480-6.

11. Seo, T., P. Pahwa, H.H. McDuffie, K. Yurube, M. Egoshi, Y. Umemoto, S. Ghosh, Y. Fukushima, and K. Nakagawa, *Association between cytochrome P450 3A5 polymorphism and the lung function in Saskatchewan grain workers.* Pharmacogenet Genomics, 2008. **18**(6): p. 487-93.

12. Calikoglu, M., L. Tamer, N. Ates Aras, S. Karakas, and B. Ercan, *The association between polymorphic genotypes of glutathione S-transferases and COPD in the Turkish population.* Biochem Genet, 2006. **44**(7-8): p. 307-19.

13. Arif, E., A. Vibhuti, P. Alam, D. Deepak, B. Singh, M. Athar, and M.A.Q. Pasha, *Association of CYP2E1 and NAT2 gene polymorphisms with chronic obstructive pulmonary disease.* Clin Chim Acta, 2007. **382**(1-2): p. 37-42.

14. Trajkov, D., J. Mirkovska-Stojkovikj, A. Petlichkovski, A. Strezova, O. Efinska-Mladenovska, E. Sandevska, O. Sibinovska, S. Hristomanova, E. Djulejic, J. Petrov, J. Gogusev, and M. Spiroski, *Association of cytokine gene polymorphisms with chronic obstructive pulmonary disease in Macedonians.* Iran, 2009. **8**(1): p. 31-42.

15. Yucesoy, B., M. Kurzius-Spencer, V.J. Johnson, K. Fluharty, M.L. Kashon, S. Guerra, M.I. Luster, and J.L. Burgess, *Association of cytokine gene polymorphisms with rate of decline in lung function.* J Occup Environ Med, 2008. **50**(6): p. 642-8.

16. Ishii, T., N. Keicho, S. Teramoto, A. Azuma, S. Kudoh, Y. Fukuchi, Y. Ouchi, and T. Matsuse, *Association of Gc-globulin variation with susceptibility to COPD and diffuse panbronchiolitis.* Eur Respir J, 2001. **18**(5): p. 753-7.

17. Jiang, L., B. He, M.-W. Zhao, L.-D. Ning, X.-Y. Li, and W.-Z. Yao, *Association of gene polymorphisms of tumour necrosis factor-alpha and interleukin-13 with chronic obstructive pulmonary disease in Han nationality in Beijing.* Chin Med J, 2005. **118**(7): p. 541-7.

18. He, J.Q., K. Shumansky, J.E. Connett, N.R. Anthonisen, P.D. Pare, and A.J. Sandford, *Association of genetic variations in the CSF2 and CSF3 genes with lung function in smoking-induced COPD.* Eur Respir J, 2008. **32**(1): p. 25-34.

19. Matheson, M.C., J.A. Ellis, J. Raven, E.H. Walters, and M.J. Abramson, *Association of IL8, CXCR2 and TNF-alpha polymorphisms and airway disease.* J Hum Genet, 2006. **51**(3): p. 196-203.

20. Cordoba-Lanus, E., J.-P. de-Torres, C. Lopez-Aguilar, M.-C. Rodriguez-Perez, N. Maca-Meyer, A. Montejo-de-Garcini, A. Aguirre-Jaime, L. Perez-Mendez, and C. Casanova, *Association of IL-6 gene polymorphisms and COPD in a Spanish population.* Respir Med, 2008. **102**(12): p. 1805-11.

21. Kang, M.-J., S.-Y. Lee, H.-B. Kim, J. Yu, B.-J. Kim, W.-A. Choi, S.-O. Jang, and S.-J. Hong, *Association of IL-13 polymorphisms with leukotriene receptor antagonist drug responsiveness in Korean children with exercise-induced bronchoconstriction.* Pharmacogenet Genomics, 2008. **18**(7): p. 551-8.

22. Huang, N., L. Liu, X.-Z. Wang, D. Liu, S.-Y. Yin, and X.-D. Yang, *Association of interleukin (IL)-12 and IL-27 gene polymorphisms with chronic obstructive pulmonary disease in a Chinese population.* DNA Cell Biol, 2008. **27**(9): p. 527-31.

23. Homma, S., T. Sakamoto, A.E. Hegab, W. Saitoh, A. Nomura, Y. Ishii, Y. Morishima, T. Iizuka, T. Kiwamoto, Y. Matsuno, H.H. Massoud, H.M. Massoud, K.M. Hassanein, and K. Sekizawa, *Association of phosphodiesterase 4D gene polymorphisms with chronic obstructive pulmonary disease: relationship to interleukin 13 gene polymorphism.* Int J Mol Med, 2006. **18**(5): p. 933-9.

24. Smith, A.K., L.A. Lange, E.J. Ampleford, D.A. Meyers, E.R. Bleecker, and T.D. Howard, *Association of polymorphisms in CASP10 and CASP8 with FEV(1)/FVC and bronchial hyperresponsiveness in ethnically diverse asthmatics.* Clin Exp Allergy, 2008. **38**(11): p. 1738-44.

25. Rohde, G., W. Klein, U. Arinir, M. Hagedorn, N. Duerig, T. T Bauer, A. Gillissen, G. Schultze-Werninghaus, and J. T Epplen, *Association of the ASP299GLY TLR4 polymorphism with COPD.* Respir Med, 2006. **100**(5): p. 892-6.

26. Ito, M., M. Hanaoka, Y. Droma, O. Hatayama, E. Sato, Y. Katsuyama, K. Fujimoto, and M. Ota, *The association of transforming growth factor beta 1 gene polymorphisms with the emphysema phenotype of COPD in Japanese.* Intern Med, 2008. **47**(15): p. 1387-94.

27. Zhu, G., I. Investigators, A. Gulsvik, P. Bakke, S. Ghatta, W. Anderson, D.A. Lomas, E.K. Silverman, and S.G. Pillai, *Association of TRPV4 gene polymorphisms with chronic obstructive pulmonary disease.* Hum Mol Genet, 2009. **18**(11): p. 2053-62.

28. Sakao, S., K. Tatsumi, H. Igari, Y. Shino, H. Shirasawa, and T. Kuriyama, *Association of tumor necrosis factor alpha gene promoter polymorphism with the presence of chronic obstructive pulmonary disease.* Am J Respir Crit Care Med, 2001. **163**(2): p. 420-2.

29. Yang, I.A., O. Holz, R.A. Jorres, H. Magnussen, S.J. Barton, S. Rodriguez, J.A. Cakebread, J.W. Holloway, and S.T. Holgate, *Association of tumor necrosis factor-alpha polymorphisms and ozone-induced change in lung function.* Am J Respir Crit Care Med, 2005. **171**(2): p. 171-6.

30. Sharma, S., A.J. Murphy, M.E. Soto-Quiros, L. Avila, B.J. Klanderman, J.S. Sylvia, J.C. Celedon, B.A. Raby, and S.T. Weiss, *Association of VEGF polymorphisms with childhood asthma, lung function and airway responsiveness.* Eur Respir J, 2009. **33**(6): p. 1287-94.

31. Raby, B.A., R. Lazarus, E.K. Silverman, S. Lake, C. Lange, M. Wjst, and S.T. Weiss, *Association of vitamin D receptor gene polymorphisms with childhood and adult asthma.* Am J Respir Crit Care Med, 2004. **170**(10): p. 1057-65.

32. Hersh, C.P., D.L. Demeo, C. Lange, A.A. Litonjua, J.J. Reilly, D. Kwiatkowski, N. Laird, J.S. Sylvia, D. Sparrow, F.E. Speizer, S.T. Weiss, and E.K. Silverman, *Attempted replication of reported chronic obstructive pulmonary disease candidate gene associations.* Am J Respir Cell Mol Biol, 2005. **33**(1): p. 71-8.

33. Turner, S.W., S.K. Khoo, I.A. Laing, L.J. Palmer, N.A. Gibson, P. Rye, L.I. Landau, J. Goldblatt, and P.N. Le Souef, *beta2 adrenoceptor Arg16Gly polymorphism, airway responsiveness, lung function and asthma in infants and children.* Clin Exp Allergy, 2004. **34**(7): p. 1043-8.

34. Summerhill, E., S.A. Leavitt, H. Gidley, R. Parry, J. Solway, and C. Ober, *beta(2)-adrenergic receptor Arg16/Arg16 genotype is associated with reduced lung function, but not with asthma, in the Hutterites.* Am J Respir Crit Care Med, 2000. **162**(2 Pt 1): p. 599-602.

35. van der Pouw Kraan, T.C.T.M., M. Kucukaycan, A.M. Bakker, J.M.C. Baggen, J.S. van der Zee, M.A. Dentener, E.F.M. Wouters, and C.L. Verweij, *Chronic obstructive pulmonary disease is associated with the -1055 IL-13 promoter polymorphism.* Genes Immun, 2002. **3**(7): p. 436-9.

36. Hu, R.-C., Y.-J. Xu, Z.-X. Zhang, W. Ni, and S.-X. Chen, *Correlation of HDEFB1 polymorphism and susceptibility to chronic obstructive pulmonary disease in Chinese Han population.* Chin Med J, 2004. **117**(11): p. 1637-41.

37. Arif, E., A. Vibhuti, D. Deepak, B. Singh, M.S. Siddiqui, and M.A.Q. Pasha, *COX2 and p53 risk-alleles coexist in COPD.* Clin Chim Acta, 2008. **397**(1-2): p. 48-50.

38. Hong, X., H. Zhou, H.J. Tsai, X. Wang, X. Liu, B. Wang, and X. Xu, *Cysteinyl leukotriene receptor 1 gene variation and risk of asthma.* Eur Respir J, 2009. **33**(1): p. 42-8.

39. van Diemen, C.C., D.S. Postma, J.M. Vonk, M. Bruinenberg, I.M. Nolte, and H.M. Boezen, *Decorin and TGF-beta1 polymorphisms and development of COPD in a general population.* Respir Res, 2006. **7**: p. 89.

40. Ober, C., Z. Tan, Y. Sun, J.D. Possick, L. Pan, R. Nicolae, S. Radford, R.R. Parry, A. Heinzmann, K.A. Deichmann, L.A. Lester, J.E. Gern, R.F. Lemanske, Jr., D.L. Nicolae, J.A. Elias, and G.L. Chupp, *Effect of variation in CHI3L1 on serum YKL-40 level, risk of asthma, and lung function.* N Engl J Med, 2008. **358**(16): p. 1682-91.

41. Arif, E., A. Ahsan, A. Vibhuti, C. Rajput, D. Deepak, M. Athar, B. Singh, and M.A.Q. Pasha, *Endothelial nitric oxide synthase gene variants contribute to oxidative stress in COPD.* Biochem Biophys Res Commun, 2007. **361**(1): p. 182-8.

42. Dijkstra, A., T.D. Howard, J.M. Vonk, E.J. Ampleford, L.A. Lange, E.R. Bleecker, D.A. Meyers, and D.S. Postma, *Estrogen receptor 1 polymorphisms are associated with airway hyperresponsiveness and lung function decline, particularly in female subjects with asthma.* J Allergy Clin Immunol, 2006. **117**(3): p. 604-11.

43. Taille, C., A. Guenegou, A. Almolki, M. Piperaud, B. Leynaert, S. Vuillaumier, F. Neukirch, J. Boczkowski, M. Aubier, J. Benessiano, and B. Crestani, *ETB receptor polymorphism is associated with airway obstruction.* BMC polm, 2007. **7**: p. 5.

44. Randolph, A.G., C. Lange, E.K. Silverman, R. Lazarus, and S.T. Weiss, *Extended haplotype in the tumor necrosis factor gene cluster is associated with asthma and asthma-related phenotypes.* Am J Respir Crit Care Med, 2005. **172**(6): p. 687-92.

45. Silverman, E.K., D.J. Kwiatkowski, J.S. Sylvia, R. Lazarus, J.M. Drazen, C. Lange, N.M. Laird, and S.T. Weiss, *Family-based association analysis of beta2-adrenergic receptor polymorphisms in the childhood asthma management program.* J Allergy Clin Immunol, 2003. **112**(5): p. 870-6.

46. Wilk, J.B., R.E. Walter, J.M. Laramie, D.J. Gottlieb, and G.T. O'Connor, *Framingham Heart Study genome-wide association: results for pulmonary function measures.* BMC Med Genet, 2007. **8 Suppl 1**: p. S8.

47. Young, R.P., R. Hopkins, P.N. Black, C. Eddy, L. Wu, G.D. Gamble, G.D. Mills, J.E. Garrett, T.E. Eaton, and M.I. Rees, *Functional variants of antioxidant genes in smokers with COPD and in those with normal lung function.* Thorax, 2006. **61**(5): p. 394-9.

48. Brogger, J., V.M. Steen, H.G. Eiken, A. Gulsvik, and P. Bakke, *Genetic association between COPD and polymorphisms in TNF, ADRB2 and EPHX1.* Eur Respir J, 2006. **27**(4): p. 682-8.

49. Cheng, S.L., C.J. Yu, C.J. Chen, and P.C. Yang, *Genetic polymorphism of epoxide hydrolase and glutathione S-transferase in COPD.* Eur Respir J, 2004. **23**(6): p. 818-24.

50. Vibhuti, A., E. Arif, D. Deepak, B. Singh, and M.A. Qadar Pasha, *Genetic polymorphisms of GSTP1 and mEPHX correlate with oxidative stress markers and lung function in COPD.* Biochem Biophys Res Commun, 2007. **359**(1): p. 136-42.

51. Matsushita, I., K. Hasegawa, K. Nakata, K. Yasuda, K. Tokunaga, and N. Keicho, *Genetic variants of human beta-defensin-1 and chronic obstructive pulmonary disease.* Biochem Biophys Res Commun, 2002. **291**(1): p. 17-22.

52. Park, H.-W., J.-E. Lee, S.-H. Kim, Y.-K. Kim, K.-U. Min, Y.-Y. Kim, and S.-H. Cho, *Genetic variation of IL13 as a risk factor of reduced lung function in children and adolescents: a cross-sectional population-based study in Korea.* Respir Med, 2009. **103**(2): p. 284-8.

53. Pillai, S.G., D. Ge, G. Zhu, X. Kong, K.V. Shianna, A.C. Need, S. Feng, C.P. Hersh, P. Bakke, A. Gulsvik, A. Ruppert, K.C. Lodrup Carlsen, A. Roses, W. Anderson, S.I. Rennard, D.A. Lomas, E.K. Silverman, D.B. Goldstein, and I. Investigators, *A genome-wide association study in chronic obstructive pulmonary disease (COPD): identification of two major susceptibility loci.* PLoS Genet, 2009. **5**(3): p. e1000421.

54. Wilk, J.B., T.-H. Chen, D.J. Gottlieb, R.E. Walter, M.W. Nagle, B.J. Brandler, R.H. Myers, I.B. Borecki, E.K. Silverman, S.T. Weiss, and G.T. O'Connor, *A genome-wide association study of pulmonary function measures in the Framingham Heart Study.* PLoS Genet, 2009. **5**(3): p. e1000429.

55. Tesfaigzi, Y., O.B. Myers, C.A. Stidley, K. Schwalm, M. Picchi, R.E. Crowell, F.D. Gilliland, and S.A. Belinsky, *Genotypes in matrix metalloproteinase 9 are a risk factor for COPD.* Int J Chron Obstruct Pulmon Dis, 2006. **1**(3): p. 267-78.

56. Corvol, H., N. Nathan, C. Charlier, K. Chadelat, P. Le Rouzic, O. Tabary, B. Fauroux, A. Henrion-Caude, J. Feingold, P.-Y. Boelle, and A. Clement, *Glucocorticoid receptor gene polymorphisms associated with progression of lung disease in young patients with cystic fibrosis.* Respir Res, 2007. **8**: p. 88.

57. Hawkins, G.A., R. Lazarus, R.S. Smith, K.G. Tantisira, D.A. Meyers, S.P. Peters, S.T. Weiss, and E.R. Bleecker, *The glucocorticoid receptor heterocomplex gene STIP1 is associated with improved lung function in asthmatic subjects treated with inhaled corticosteroids.* J Allergy Clin Immunol, 2009. **123**(6): p. 1376-83.e7.

58. Imboden, M., S.H. Downs, O. Senn, G. Matyas, O. Brandli, E.W. Russi, C. Schindler, U. Ackermann-Liebrich, W. Berger, N.M. Probst-Hensch, and S. Team, *Glutathione S-transferase genotypes modify lung function decline in the general population: SAPALDIA cohort study.* Respir Res, 2007. **8**: p. 2.

59. Ishii, T., T. Matsuse, S. Teramoto, H. Matsui, M. Miyao, T. Hosoi, H. Takahashi, Y. Fukuchi, and Y. Ouchi, *Glutathione S-transferase P1 (GSTP1) polymorphism in patients with chronic obstructive pulmonary disease.* Thorax, 1999. **54**(8): p. 693-6.

60. Rodriguez, F., C. de la Roza, R. Jardi, M. Schaper, R. Vidal, and M. Miravitlles, *Glutathione S-transferase P1 and lung function in patients with alpha1-antitrypsin deficiency and COPD.* Chest, 2005. **127**(5): p. 1537-43.

61. Yang, S.-f., Y.-j. Xu, J.-g. Xie, and Z.-x. Zhang, *hOGG1 Ser326Cys and XRCC1 Arg399Gln polymorphisms associated with chronic obstructive pulmonary disease.* Chin Med J, 2009. **122**(8): p. 960-6.

62. Yanbaeva, D.G., M.A. Dentener, M.A. Spruit, J.J. Houwing-Duistermaat, D. Kotz, V.L. Passos, and E.F.M. Wouters, *IL6 and CRP haplotypes are associated with COPD risk and systemic inflammation: a case-control study.* BMC Med Genet, 2009. **10**: p. 23.

63. Lyon, H., C. Lange, S. Lake, E.K. Silverman, A.G. Randolph, D. Kwiatkowski, B.A. Raby, R. Lazarus, K.M. Weiland, N. Laird, and S.T. Weiss, *IL10 gene polymorphisms are associated with asthma phenotypes in children.* Genet Epidemiol, 2004. **26**(2): p. 155-65.

64. Demeo, D.L., E.J. Campbell, A.F. Barker, M.L. Brantly, E. Eden, N.G. McElvaney, S.I. Rennard, R.A. Sandhaus, J.M. Stocks, J.K. Stoller, C. Strange, G. Turino, and E.K. Silverman, *IL10 polymorphisms are associated with airflow obstruction in severe alpha1-antitrypsin deficiency.* Am J Respir Cell Mol Biol, 2008. **38**(1): p. 114-20.

65. Hong, S.-J., S.-Y. Lee, H.-B. Kim, J.-H. Kim, B.-S. Kim, S.-O. Choi, S.-G. Lee, E.-S. Shin, and T.-J. Hong, *IL-5 and thromboxane A2 receptor gene polymorphisms are associated with decreased pulmonary function in Korean children with atopic asthma.* J Allergy Clin Immunol, 2005. **115**(4): p. 758-63.

66. Lima, J.J., S. Zhang, A. Grant, L. Shao, K.G. Tantisira, H. Allayee, J. Wang, J. Sylvester, J. Holbrook, R. Wise, S.T. Weiss, and K. Barnes, *Influence of leukotriene pathway polymorphisms on response to montelukast in asthma.* Am J Respir Crit Care Med, 2006. **173**(4): p. 379-85.

67. He, J.-Q., K. Burkett, J.E. Connett, N.R. Anthonisen, P.D. Pare, and A.J. Sandford, *Interferon gamma polymorphisms and their interaction with smoking are associated with lung function.* Hum Genet, 2006. **119**(4): p. 365-75.

68. Sata, M., N. Takabatake, S. Inoue, Y. Shibata, S. Abe, J.-i. Machiya, T. Wada, G. Ji, T. Kido, T. Matsuura, M.-a. Muramatsu, and I. Kubota, *Intronic single-nucleotide polymorphisms in Bcl-2 are associated with chronic obstructive pulmonary disease severity.* Respirology, 2007. **12**(1): p. 34-41.

69. Hansel, N.N., L. Gao, N.M. Rafaels, R.A. Mathias, E.R. Neptune, C. Tankersley, A.V. Grant, J. Connett, T.H. Beaty, R.A. Wise, and K.C. Barnes, *Leptin receptor polymorphisms and lung function decline in COPD.* Eur Respir J, 2009. **34**(1): p. 103-10.

70. Young, R.P., R.J. Hopkins, B.A. Hay, M.J. Epton, P.N. Black, and G.D. Gamble, *Lung cancer gene associated with COPD: triple whammy or possible confounding effect?* Eur Respir J, 2008. **32**(5): p. 1158-64.

71. Yoshikawa, M., K. Hiyama, S. Ishioka, H. Maeda, A. Maeda, and M. Yamakido, *Microsomal epoxide hydrolase genotypes and chronic obstructive pulmonary disease in Japanese.* Int J Mol Med, 2000. **5**(1): p. 49-53.

72. Takabatake, N., S. Toriyama, Y. Takeishi, Y. Shibata, T. Konta, S. Inoue, S. Abe, A. Igarashi, Y. Tokairin, M. Ishii, S. Koyano, M. Emi, T. Kato, S. Kawata, and I. Kubota, *A nonfunctioning single nucleotide polymorphism in olfactory receptor gene family is associated with the forced expiratory volume in the first second/the forced vital capacity values of pulmonary function test in a Japanese population.* Biochem Biophys Res Commun, 2007. **364**(3): p. 662-7.

73. Galanter, J., S. Choudhry, C. Eng, S. Nazario, J.R. Rodriguez-Santana, J. Casal, A. Torres-Palacios, J. Salas, R. Chapela, H.G. Watson, K. Meade, M. LeNoir, W. Rodriguez-Cintron, P.C. Avila, and E.G. Burchard, *ORMDL3 gene is associated with asthma in three ethnically diverse populations.* Am J Respir Crit Care Med, 2008. **177**(11): p. 1194-200.

74. Barton, S.J., G.H. Koppelman, J.M. Vonk, C.A. Browning, I.M. Nolte, C.E. Stewart, S. Bainbridge, S. Mutch, M.J. Rose-Zerilli, D.S. Postma, N. Maniatis, A.P. Henry, I.P. Hall, S.T. Holgate, P. Tighe, J.W. Holloway, and I. Sayers, *PLAUR polymorphisms are associated with asthma, PLAUR levels, and lung function decline.* J Allergy Clin Immunol, 2009. **123**(6): p. 1391-400.e17.

75. Park, J.Y., L. Chen, N. Wadhwa, and M.S. Tockman, *Polymorphisms for microsomal epoxide hydrolase and genetic susceptibility to COPD.* Int J Mol Med, 2005. **15**(3): p. 443-8.

76. Simpson, A., N. Maniatis, F. Jury, J.A. Cakebread, L.A. Lowe, S.T. Holgate, A. Woodcock, W.E.R. Ollier, A. Collins, A. Custovic, J.W. Holloway, and S.L. John, *Polymorphisms in a disintegrin and metalloprotease 33 (ADAM33) predict impaired early-life lung function.* Am J Respir Crit Care Med, 2005. **172**(1): p. 55-60.

77. Lee, J.M., Y.R. Kang, S.H. Park, S.I. Cha, J.S. Kim, H.K. Kang, W.K. Lee, M.J. Kim, C.H. Kim, N.S. Kim, T.H. Jung, and J.Y. Park, *Polymorphisms in interleukin-1B and its receptor antagonist genes and the risk of chronic obstructive pulmonary disease in a Korean population: a case-control study.* Respir Med, 2008. **102**(9): p. 1311-20.

78. Litonjua, A.A., K.G. Tantisira, S. Lake, R. Lazarus, B.G. Richter, S. Gabriel, E.S. Silverman, and S.T. Weiss, *Polymorphisms in signal transducer and activator of transcription 3 and lung function in asthma.* Respir Res, 2005. **6**: p. 52.

79. Joos, L., T.D. Weir, J.E. Connett, N.R. Anthonisen, R. Woods, P.D. Pare, and A.J. Sandford, *Polymorphisms in the beta2 adrenergic receptor and bronchodilator response, bronchial hyperresponsiveness, and rate of decline in lung function in smokers.* Thorax, 2003. **58**(8): p. 703-7.

80. LeVan, T.D., S. Von Essen, D.J. Romberger, G.P. Lambert, F.D. Martinez, M.M. Vasquez, and J.A. Merchant, *Polymorphisms in the CD14 gene associated with pulmonary function in farmers.* Am J Respir Crit Care Med, 2005. **171**(7): p. 773-9.

81. Kim, K.M., S.H. Park, J.S. Kim, W.K. Lee, S.I. Cha, C.H. Kim, Y.M. Kang, T.H. Jung, I.S. Kim, and J.Y. Park, *Polymorphisms in the type IV collagen alpha3 gene and the risk of COPD.* Eur Respir J, 2008. **32**(1): p. 35-41.

82. Hegab, A.E., T. Sakamoto, W. Saitoh, H.H. Massoud, H.M. Massoud, K.M. Hassanein, and K. Sekizawa, *Polymorphisms of IL4, IL13, and ADRB2 genes in COPD.* Chest, 2004. **126**(6): p. 1832-9.

83. Lee, Y.-L., W. Chen, W.-K. Tsai, J.-C. Lee, H.-L. Chiou, C.-M. Shih, and Y.-C. Wang, *Polymorphisms of p53 and p21 genes in chronic obstructive pulmonary disease.* J Lab Clin Med, 2006. **147**(5): p. 228-33.

84. Hegab, A.E., T. Sakamoto, W. Saitoh, A. Nomura, Y. Ishii, Y. Morishima, T. Iizuka, T. Kiwamoto, Y. Matsuno, H.H. Massoud, H.M. Massoud, K.M. Hassanein, and K. Sekizawa, *Polymorphisms of TNFalpha, IL1beta, and IL1RN genes in chronic obstructive pulmonary disease.* Biochem Biophys Res Commun, 2005. **329**(4): p. 1246-52.

85. Hegab, A.E., T. Sakamoto, Y. Uchida, A. Nomura, Y. Ishii, Y. Morishima, M. Mochizuki, T. Kimura, W. Saitoh, T. Iizuka, T. Kiwamoto, and K. Sekizawa, *Promoter activity of human tissue inhibitor of metalloproteinase 2 gene with novel single nucleotide polymorphisms.* Respirology, 2005. **10**(1): p. 27-30.

86. Tsai, Y.J., S. Choudhry, J. Kho, K. Beckman, H.-J. Tsai, D. Navarro, H. Matallana, R.A. Castro, C.M. Lilly, S. Nazario, J.R. Rodriguez-Santana, J. Casal, A. Torres, J. Salas, R. Chapela, H.G. Watson, K. Meade, P.C. Avila, W. Rodriguez-Cintron, M. LeNoir, E.G. Burchard, S. Genetics of Asthma in Latino Americans, A.G. Study of African Americans, and I. Environments, *The PTGDR gene is not associated with asthma in 3 ethnically diverse populations.* J Allergy Clin Immunol, 2006. **118**(6): p. 1242-8.

87. Shen, M., R. Vermeulen, R.S. Chapman, S.I. Berndt, X. He, S. Chanock, N. Caporaso, and Q. Lan, *A report of cytokine polymorphisms and COPD risk in Xuan Wei, China.* Int J Hyg Environ Health, 2008. **211**(3-4): p. 352-6.

88. Ito, I., S. Nagai, Y. Hoshino, S. Muro, T. Hirai, M. Tsukino, and M. Mishima, *Risk and severity of COPD is associated with the group-specific component of serum globulin 1F allele.* Chest, 2004. **125**(1): p. 63-70.

89. Holloway, J.W., S.J. Barton, S.T. Holgate, M.J. Rose-Zerilli, and I. Sayers, *The role of LTA4H and ALOX5AP polymorphism in asthma and allergy susceptibility.* Allergy, 2008. **63**(8): p. 1046-53.

90. Joos, L., J.-Q. He, M.B. Shepherdson, J.E. Connett, N.R. Anthonisen, P.D. Pare, and A.J. Sandford, *The role of matrix metalloproteinase polymorphisms in the rate of decline in lung function.[Erratum appears in Hum Mol Genet. 2003 Apr 1;12(7):803-4].* Hum Mol Genet, 2002. **11**(5): p. 569-76.

91. Schedel, M., M. Depner, C. Schoen, S.K. Weiland, C. Vogelberg, B. Niggemann, S. Lau, T. Illig, N. Klopp, U. Wahn, E. von Mutius, R. Nickel, and M. Kabesch, *The role of polymorphisms in ADAM33, a disintegrin and metalloprotease 33, in childhood asthma and lung function in two German populations.* Respir Res, 2006. **7**: p. 91.

92. Wilk, J.B., A. Herbert, C.M. Shoemaker, D.J. Gottlieb, and S. Karamohamed, *Secreted modular calcium-binding protein 2 haplotypes are associated with pulmonary function.* Am J Respir Crit Care Med, 2007. **175**(6): p. 554-60.

93. DeMeo, D., T. Mariani, C. Lange, S. Lake, A. Litonjua, J. Celedon, J. Reilly, H.A. Chapman, D. Sparrow, A. Spira, J. Beane, V. Pinto-Plata, F.E. Speizer, S. Shapiro, S.T. Weiss, and E.K. Silverman, *The SERPINE2 gene is associated with chronic obstructive pulmonary disease.* Proc, 2006. **3**(6): p. 502.

94. Zhu, G., L. Warren, J. Aponte, A. Gulsvik, P. Bakke, W.H. Anderson, D.A. Lomas, E.K. Silverman, S.G. Pillai, and C.G.N.I. International, *The SERPINE2 gene is associated with chronic obstructive pulmonary disease in two large populations.* Am J Respir Crit Care Med, 2007. **176**(2): p. 167-73.

95. Sandford, A.J., T. Chagani, T.D. Weir, J.E. Connett, N.R. Anthonisen, and P.D. Pare, *Susceptibility genes for rapid decline of lung function in the lung health study.* Am J Respir Crit Care Med, 2001. **163**(2): p. 469-73.

96. Sunyer, J., R. Pistelli, E. Plana, M. Andreani, F. Baldari, M. Kolz, W. Koenig, J. Pekkanen, A. Peters, and F. Forastiere, *Systemic inflammation, genetic susceptibility and lung function.* Eur Respir J, 2008. **32**(1): p. 92-7.

97. Raby, B.A., E.-S. Hwang, K. Van Steen, K. Tantisira, S. Peng, A. Litonjua, R. Lazarus, C. Giallourakis, J.D. Rioux, D. Sparrow, E.K. Silverman, L.H. Glimcher, and S.T. Weiss, *T-bet polymorphisms are associated with asthma and airway hyperresponsiveness.* Am J Respir Crit Care Med, 2006. **173**(1): p. 64-70.

98. Arkwright, P.D., S. Laurie, M. Super, V. Pravica, M.J. Schwarz, A.K. Webb, and I.V. Hutchinson, *TGF-beta(1) genotype and accelerated decline in lung function of patients with cystic fibrosis.* Thorax, 2000. **55**(6): p. 459-62.

99. Leung, T.F., N.L.S. Tang, C.W.K. Lam, A.M. Li, I.H.S. Chan, and G. Ha, *Thromboxane A2 receptor gene polymorphism is associated with the serum concentration of cat-specific immunoglobulin E as well as the development and severity of asthma in Chinese children.* Pediatr Allergy Immunol, 2002. **13**(1): p. 10-7.

100. Zhang, H., J. Hang, X. Wang, W. Zhou, B. Sun, H. Dai, L. Su, and D.C. Christiani, *TNF polymorphisms modify endotoxin exposure-associated longitudinal lung function decline.* Occup Environ Med, 2007. **64**(6): p. 409-13.

101. Celedon, J.C., C. Lange, B.A. Raby, A.A. Litonjua, L.J. Palmer, D.L. DeMeo, J.J. Reilly, D.J. Kwiatkowski, H.A. Chapman, N. Laird, J.S. Sylvia, M. Hernandez, F.E. Speizer, S.T. Weiss, and E.K. Silverman, *The transforming growth factor-beta1 (TGFB1) gene is associated with chronic obstructive pulmonary disease (COPD).* Hum Mol Genet, 2004. **13**(15): p. 1649-56.

102. Hersh, C.P., N.N. Hansel, K.C. Barnes, D.A. Lomas, S.G. Pillai, H.O. Coxson, R.A. Mathias, N.M. Rafaels, R.A. Wise, J.E. Connett, B.J. Klanderman, F.L. Jacobson, R. Gill, A.A. Litonjua, D. Sparrow, J.J. Reilly, E.K. Silverman, and I. Investigators, *Transforming growth factor-beta receptor-3 is associated with pulmonary emphysema.* Am J Respir Cell Mol Biol, 2009. **41**(3): p. 324-31.

103. Ruse, C.E., M.C. Hill, M. Tobin, N. Neale, M.J. Connolly, S.G. Parker, and A.J. Wardlaw, *Tumour necrosis factor gene complex polymorphisms in chronic obstructive pulmonary disease.* Respir Med, 2007. **101**(2): p. 340-4.

104. Schellenberg, D., P.D. Pare, T.D. Weir, J.J. Spinelli, B.A. Walker, and A.J. Sandford, *Vitamin D binding protein variants and the risk of COPD.* Am J Respir Crit Care Med, 1998. **157**(3 Pt 1): p. 957-61.
